# Gd-Si Oxide Nanoparticles as Contrast Agents in Magnetic Resonance Imaging

**DOI:** 10.3390/nano6060109

**Published:** 2016-06-08

**Authors:** Alejandro Cabrera-García, Alejandro Vidal-Moya, Ángela Bernabeu, Jesús Pacheco-Torres, Elisa Checa-Chavarria, Eduardo Fernández, Pablo Botella

**Affiliations:** 1 Instituto de Tecnología Química, Universitat Politècnica de València-Consejo Superior de Investigaciones Científicas, Avenida de los Naranjos s/n, 46022 Valencia, Spain; alcabgar@itq.upv.es (A.C.-G.); svidal@itq.upv.es (A.V.-M.); 2 Unit of Magnetic Resonance Imaging, Hospital Universitario de Alicante, INSCANNER S.L., Alicante, Spain; angela.bernabeu@gmail.com; 3 Unit of Functional Magnetic Resonance Imaging, Instituto de Neurociencias (CSIC-UMH), Alicante, Spain; jpacheco@umh.es; 4 Institute of Bioengineering, Universidad Miguel Hernández, Elche, Spain and Centre for Network Biomedical Research (CIBER-BBN), Spain; echeca@goumh.umh.es (E.C.-C.); e.fernandez@umh.es (E.F.)

**Keywords:** Prussian Blue analogues, Gd-Si nanoparticles, magnetic resonance imaging, contrast agent

## Abstract

We describe the synthesis, characterization and application as contrast agents in magnetic resonance imaging of a novel type of magnetic nanoparticle based on Gd-Si oxide, which presents high Gd^3+^ atom density. For this purpose, we have used a Prussian Blue analogue as the sacrificial template by reacting with soluble silicate, obtaining particles with nanorod morphology and of small size (75 nm). These nanoparticles present good biocompatibility and higher longitudinal and transversal relaxivity values than commercial Gd^3+^ solutions, which significantly improves the sensitivity of *in vivo* magnetic resonance images.

## 1. Introduction

One of the main reasons why cancer is a major medical challenge of the 21st century is that, in many cases, therapy success is mostly affected by timely diagnosis and prognosis. In this sense, it is crucial to find novel approaches of diagnostic techniques capable of detecting the malignancy at early stages. Here, magnetic resonance imaging (MRI) is a non-invasive technique in medical research that presents several advantages (use of non-ionizing radiation, widespread clinical implementation, and good anatomical resolution) that make it one of the most powerful imaging techniques [1,2,3]. Contrast agents (CA) are used for multiple purposes in MRI, one of them being the clinical detection of tumors [4]. Based on their relaxation processes, CAs are classified as *T*_1_-positive agents of paramagnetic species and *T*_2_-negative agents of super-paramagnetic materials. Due to their high sensitivity and detectability, Gd^3+^-based soluble chelates are the most representative and preferential *T*_1_ CAs in current MRI clinical applications [5]. However, their poor pharmacokinetics profile, due to quick renal filtration, limits the sensitivity [6]. This, consequently, requires the administration of generous CA doses, e.g., perfusion studies for tumor detection, which may lead to some side effects such as headache, nausea, and dizziness [7]. Among the most significant side effects reported regarding the administration of Gd^3+^ compounds, we find nephrogenic systemic fibrosis in patients with impaired kidney function, hypersensitivity reactions, and nephrogenic fibrosing dermopathy, which makes it expedient to develop alternatives to these small, soluble molecules [8].

At this point, magnetic nanoparticles (MNP) present several advantages over conventional Gd^3+^ CAs, such as large payloads of paramagnetic metal ions, extended plasma circulating half-lives, passive accumulation at tumor sites, and improved safety profiles [9,10]. Therefore, the incorporation of Gd^3+^ in a stable nanocarrier points to the development of safe and efficient CAs for MRI. In this context, silica nanomaterials have been recently considered to be excellent candidates for the preparation of drug delivery systems and CAs. Firstly, their textural properties favor the loading of significant quantities of therapeutic molecules within pore channels. Secondly, it is possible to obtain nanoparticles of these materials, which results in an accurate control over pore structure, particle shape and size [11,12] and, finally, the silanol-containing surface can be easily functionalized, introducing additional features that allow imposing targeting delivery [13]. Furthermore, biocompatibility test results have shown the potential of these materials in the preparation of pharmaceutical forms that might find application in diagnostics and customized medicine [14].

The first attempt to create a silica-based Gd^3+^ CA was by incorporation, by covalent bonding or by confinement, of a Gd^3+^ chelate [15,16,17,18,19,20,21,22,23]. This way, highly paramagnetic conjugates may be obtained, in some cases with very impressive longitudinal (*r*_1_) and transversal (*r*_2_) relaxivity values. However, Gd^3+^ concentration in these conjugates is usually low, which reduces the sensitivity for *in vivo* applications. Moreover, metal leaching in biological fluids is not always negligible, which may hinder administration of recurrent doses. More stable CAs can be prepared by doping the silica matrix with Gd^3+^ cations [24,25,26,27,28,29], but the paramagnetic center density in the silicate network remains limited. A possibility to increase the number of metal centers per particle and to avoid toxicity issues is to protect small gadolinium nanoparticles with a silica shell [30,31,32,33,34,35,36,37]. These systems are very stable in physiological medium and their main drawback is the low accessibility of the Gd^3+^ sites to water molecules, due to the confining effects developed over the silica coating. It must be considered that the *r*_1_ relaxivity is highly dependent on the number of water molecules coordinating to each metal ion in the first hydration sphere (q), which plays a detrimental role in MRI properties of these materials, especially when the present trend in clinical MRI is moving towards the use of higher magnetic field strength to obtain a better signal to noise ratio (SNR).

In order to increase the number of metal centers per particle, without reducing the water accessibility, an alternative is to develop Gd silicate-based MNP, where the high ion density and a disordered structure allow for efficient interaction of most Gd^3+^ sites with the surrounding water [35]. On this basis, we introduce here a novel approach for the synthesis of Gd-Si oxide nanoparticles with high Gd^3+^ atom density and excellent stability in physiological conditions. For this purpose, we used an optimized method developed by our group [38], wherein Prussian Blue analogue (PBA) Gd(H_2_O)_4_[Fe(CN)_6_] is used as a sacrificial template by reacting with soluble silicate. By overlapping kinetics of silica condensation with [Fe(CN)_6_]^3−^ removal, we are able to synthesize particles of an oxide nanocomposite with equal shape and size than former PBA crystallites. The resulting material presents nanorod morphology, small particle size (75 nm average length), and shows good *r*_1_ and *r*_2_ relaxivity values in a 3 tesla (T) magnetic field, improving positive contrast of *in vitro* and *in vivo* MRI images.

## 2. Results and Discussion

### 2.1. Materials Preparation and Characterization

**Scheme 1:**
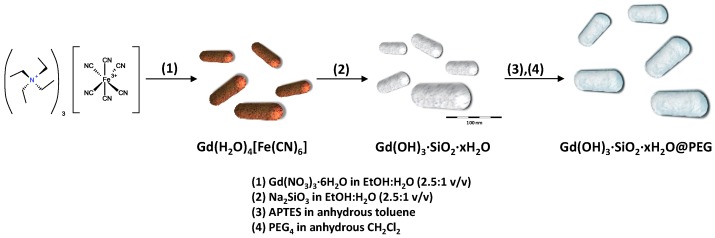
Synthesis layout of Gd-Si oxide nanorods using Gd(H_2_O)_4_[Fe(CN)_6_] as a sacrificial template.

Scheme 1 depicts the strategy to synthesize pre-formed Gd-Si oxide nanoparticles. Firstly, an improved method for the preparation of GdFe PBA with small particle size was developed.

**Figure 1:**
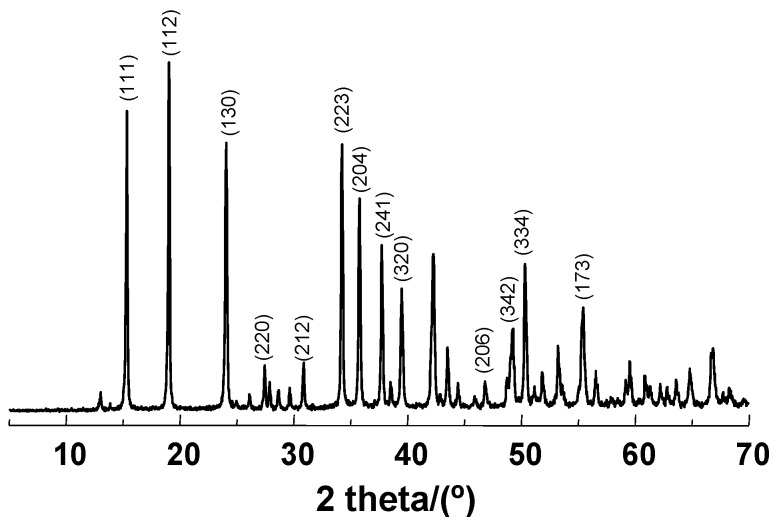
Powder X-ray diffraction (XRD) pattern of as-made GdFe nanorods.

**Figure 2:**
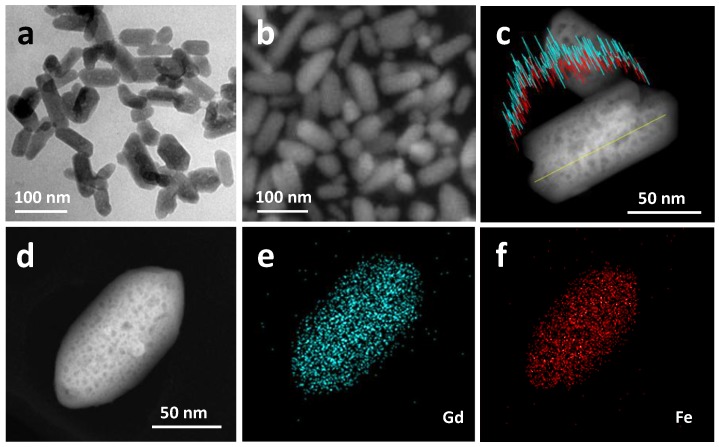
Electron microscopy study of as synthesized GdFe nanorods. (**a**,**b**): Transmission electron microscopy (TEM) (**a**) and field-emission scanning electronic microscopy (FESEM) (**b**) images; (**c**) Scanning transmission electron microscopy (STEM) image with energy-dispersive X-ray spectroscopy analysis (EDS) line scans. Legend: blue line = Gd; red line = Fe; (**d**,**f**): STEM image (**d**) and EDS mapping pictures of one GdFe nanorod (**e**,**f**).

Rod-shaped, monodispersed, high-purity particles of 94 nm average diameter were obtained (see Table 1). The crystal structure of the tridimensional coordination network Gd(H_2_O)_4_[Fe(CN)_6_] has been recently solved [39]. The solid crystallizes in the orthorhombic system with C_mcm_ space group and cell parameter of a = 7.4016(3) Å, b = 12.78813(16) Å, and c = 13.5980(12) Å. This nanosized MOF presents a fully comparable powder X-ray diffraction (XRD) pattern to that obtained for the bulk compound (Figure 1). Moreover, crystal composition, as determined by energy-dispersive X-ray spectroscopy analysis (EDS) analysis, is very homogeneous, with uniform Fe and Gd distribution within the particles, as revealed by elemental mapping results (Figure 2), which is strongly consistent with the stoichiometry of the metal-organic framework (MOF).

Such GdFe small crystallites are partially soluble in neutral water, and an ion exchange reaction takes place in alkaline solution resulting in complete MOF dissolution, as follows Equation (1):

Gd(H_2_O)_4_[Fe(CN)_6_](s) + 3OH^−^(aq) ↔ Gd(OH)_3_(s) + [Fe(CN)_6_]^3−^(aq) + 4H_2_O (1)

This reaction can be carried out in the presence of silicate ions, the hydrolysis of which provides enough OH^−^ concentration for the transformation. Therefore, under optimized conditions, it is possible to overlap ion exchange of the PBA with the hydrolysis of the conjugate base, and, as a consequence, a simultaneous and stoichiometric condensation of gadolinium hydroxide and silica takes place. This process is defined by Equation (2):

2Gd(H_2_O)_4_[Fe(CN)_6_](s) + 3SiO_3_^2−^(aq) + 3(1 + *x*)H_2_O ↔ 2Gd(OH)_3_·3SiO_2_·*x*H_2_O(s) + 2[Fe(CN)_6_]^3−^(aq) + 4H_2_O (2)

Dense, monodispersed Gd-Si oxide nanoparticles (GdSi) are obtained mostly preserving the original rod shape and size of the sacrificial GdFe crystals. The reaction proceeds slowly at room temperature in a silicate solution (EtOH:H_2_O, 2.5:1 *v*/*v*). This way, MOF particles are gradually consumed, releasing Fe(CN)_6_^3−^ as hydroxide ions diffuse inward, and a composite of Gd(OH)_3_ and SiO_2_ is formed. The powder XRD pattern shows an amorphous material (figure not shown), with BET surface area of about 19.0 m^2^·g^−1^ and low porosity (Table 1). This nanohybrid low crystallinity is due to mutual inhibition of crystal growth between the highly-dispersed Gd(OH)_3_ and SiO_2_ [40].

**Figure 3:**
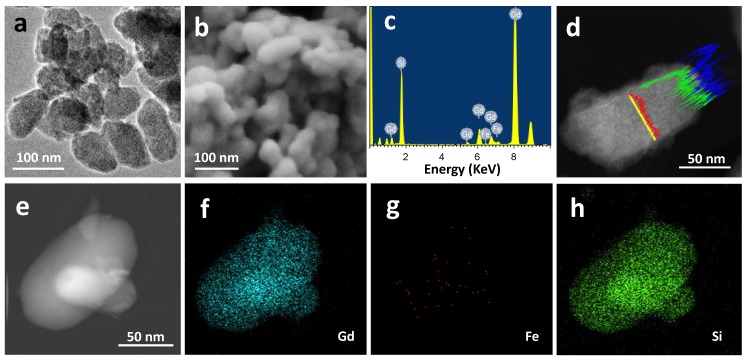
Electron microscopy study of GdSi nanorods obtained by alkaline transformation of GdFe precursor. (**a**,**b**): TEM (**a**) and FESEM (**b**) images of GdSi nanoparticles; (**c**) EDS elemental analysis; (**d**) STEM image with EDS line scans. Legend: blue line = Gd; red line = Fe; green line = Si; (**e**–**h**) STEM image (**e**) and EDS mapping pictures of two GdSi nanorods (**f**–**h**).

Electron microscopy study by TEM and FESEM (Figure 3) confirms that morphology does not experience significant changes, although nanosized rods present a slightly smaller average diameter (75 nm). As already detected with other morphologies at the nanoscale [38], our transformed particles are fully dense, what contrasts with observations by Lou and co-workers over Prusian Blue microbox derivatives [40,41]. We attribute such different material features to the nanoscale, as the inward OH^−^ ion diffusion is faster in smaller particles and 2Gd(OH)_3_·3SiO_2_·*x*H_2_O moiety precipitates quickly to refill the empty space left by released [Fe(CN)_6_]^3−^ species. EDS analysis is consistent with the expected stoichiometry (Gd:Si atomic ratio of about 2:3), and demonstrates almost complete iron removal (Table 1). Moreover, the spatial distribution of Gd(OH)_3_ and SiO_2_ in isolated particles can be clearly seen by elemental mapping (Figure 3c–h), which illustrates both the homogeneous distribution of Gd and Si, and iron extraction.

**Figure 4:**
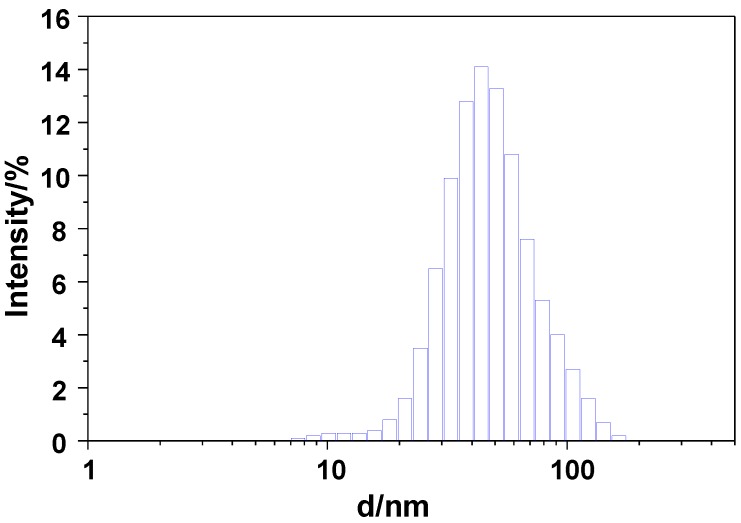
Particle hydrodynamic diameter of GdSi sample as determined in water by DLS.

The GdSi sample presents a good stability pattern for use in biological medium. In this context, the hydrodynamic diameter, as determined by diffuse light scattering (DLS) (Figure 4), is 83.9 ± 44.1 nm, is very close to that determined by TEM, and with a narrow size distribution, which indicates that most of the particles are monodispersed. Furthermore, dissolution tests carried out for 24 h in phosphate buffer saline (PBS) and in an isotonic glucose aqueous solution revealed no detectable leaching of Gd^3+^ and/or Si^4+^ cations in the incubation medium.

### 2.2. Relaxivity Measurements and In Vitro MRI

The efficacy of GdSi as *T*_1_- or *T*_2_-weighted CA for MRI was evaluated by measuring the longitudinal (*r*_1_) and transversal (*r*_2_) nuclear magnetic relaxation rates of water protons in aqueous suspensions at 3 T and room temperature. Stable colloids were prepared in aqueous xanthan gum (0.1%) solution with Gd^3+^ concentration in the range 0–1.00 mM. Relaxivity values were determined by using the following expression [42]:

(3) $$\frac{1}{T_{i\;\left(w\right)}}=\frac{1}{T_{i\left(0\right)}}+r_{i}$$

**Figure 5:**
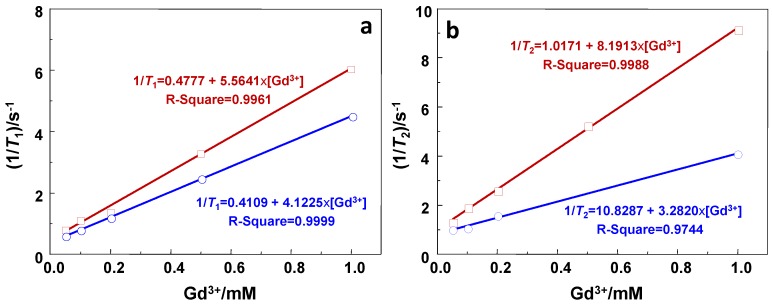
*T*_1_ (**a**) and *T*_2_ (**b**) relaxation rate measurements *vs* concentration of GdSi aqueous solutions (0.1% xanthan gum) at a magnetic field of 3 T. Legend: (□) GdSi; (◦) Gd-DTPA.

Where *i* = 1 or 2 values, respectively, for longitudinal or transversal-weighted effect of CA. 1/*T_i_*_(w)_ is the global relaxation rate constant of bulk water molecules, T*_i_*_(0)_ is the water relaxation time without contrast agents (CA), and *C* is the paramagnetic ion concentration. Therefore, *r*_1_ and *r*_2_ values were determined, respectively, from the plot slopes of 1/*T*_1_ (s^−1^) and 1/*T*_2_ (s^−1^) *versus* Gd^3+^ concentration (mM, Table 2 and Figure 5). For the sake of comparison, same measurements were carried out with commercial CA gadopentetate dihydrogen salt (Gd-DTPA, Aldrich). CAs can be classified according to their *T*_1_ or *T*_2_ lowering activity. Moreover, when *r*_2_/*r*_1_ value is close to 1, application as a positive (bright) CA is favored, whereas if this ratio increases far from 1 the CA works better for negative (dark) contrast [43].

**Figure 6:**
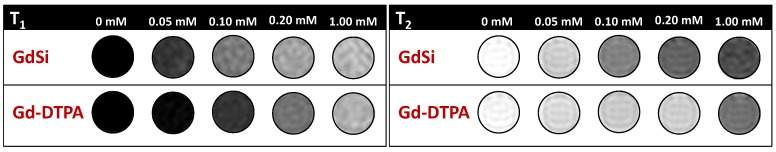
*T*_1_*-*weighted (**left**) and *T*_2_-weighted (**right**) magnetic resonance imaging (MRI) slices of GdSi aqueous solutions (0.1% xanthan gum), with varying Gd^3+^ concentration, at magnetic field of 3 T and echo time of 2 ms (*T*_1_) and 203 ms (*T*_2_).

As it can be seen in Table 2, GdSi sample shows longitudinal and transversal relaxivity values, respectively, 1.4 and 2.5 times higher than those corresponding to the soluble Gd^3+^ complex. This is in agreement with other authors’ observations for different Gd silicate nanoparticles [37]. At this point, the high Gd concentration (320 mg/g) present in this material should be considered. With regards to the mononuclear Gd^3+^ complex, such a large density of paramagnetic centers in the nanoparticle shortens *T*_1_ and *T*_2_ scores due to a cooperative effect and accessibility of all Gd^3+^ centers (surface and core) [39]. Therefore, it is noticeable that no diffusion restrictions are present between water molecules and Gd^3+^ sites located in the silicate matrix. Additionally, it must be taken into account that the presence of a small Fe^3+^ quantity in the as-synthesized GdSi sample (Table 1) might contribute to a reduced *T*_2_ value. In this sense, with a *r*_2_/*r*_1_ ratio of about 1.5, this material is expected to improve MRI images mostly in positive contrast. To further check the capability of the GdSi nanomaterial, we collected phantom MRI images (Figure 6). The phantom was scanned with a single slice in coronal orientation with variable CA or gadopentetate concentration. Results show that *T*_1_ images become progressively brighter as nanoparticle content increases (Figure 6a,b). Furthermore, *T*_2_-weighted images become darker with Gd^3+^ concentration, but with lower significance than the positive contrast (Figure 6c). In all cases, the sensitivity of this system is higher than that of the commercial Gd^3+^ chelate. This encouraged us to test these MNPs in biological conditions, as described in the next two sections.

### 2.3. Cytotoxicity Test

**Figure 7:**
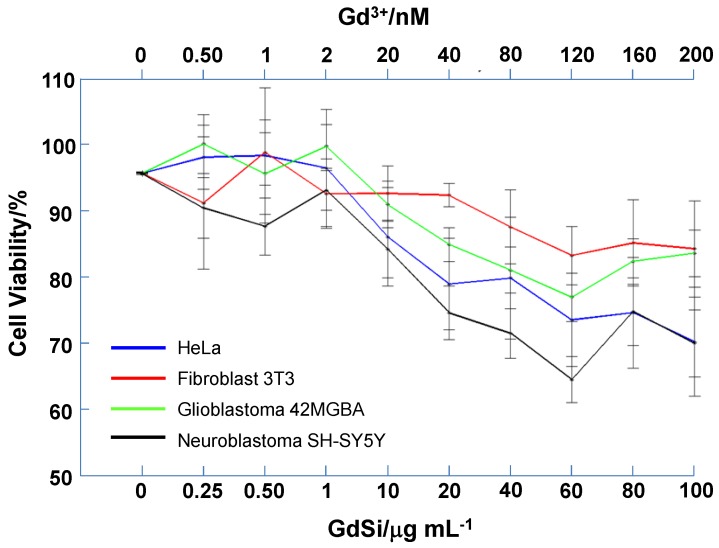
*In vitro* 3-(4,5-dimethylthiazol-2-yl)-2,5-diphenyltetrazolium bromide (MTT) cell viability assays in different cell lines incubated with variable concentrations of GdSi magnetic nanorods (mean ± SEM, *n* = 4). Top *X*-axis indicates the concentration of equivalent Gd^3+^.

In order to validate the potential performance of the Gd-Si nanoparticles in a biological environment, cell viability was assessed 24 h after incubation with the GdSi sample by measuring the conversion of 3-(4,5-dimethylthiazol-2-yl)-2,5-diphenyltetrazolium bromide (MTT) to its formazan form following standard procedures. For these experiments we used healthy fibroblasts (3T3 cell line) and several cancer cell lines (HeLa cells, 42-MG-BA glioblastoma multiforme cells and SH-SY5Y neuroblastoma cells) at a range of concentrations (0.25–100 µg·mL^−1^). Results (Figure 7) indicate that cell survival was always of about 70% or higher, even at maximum particle concentration. Despite MTT assay limitations for accurate cell viability determination, these results correspond to an acceptable biocompatibility profile.

### 2.4. In Vivo MRI Studies Imaging

It is well known that when introducing non-protected nanoparticles in blood, these suffer from strong protein adsorption, resulting in protein corona formation, which promotes further interaction with cell elements of the reticuloendothelial system (RES) [44]. As a consequence, particles are rapidly cleared from blood and accumulated in RES tissues, mainly the liver, spleen, and lungs. Consequently, for the *in vivo* study of GdSi contrast material, we firstly modified nanorod surfaces with a short polyethyleneglycol (PEG) chain, according to a recipe developed by our group [45]. This way, nanoparticles are expected to prolong circulation in the blood stream, finally being removed from the body mostly by renal (urine) and hepatic (biliary) routes [46,47]. Then, 1.0 mL of a 5 mg·mL^−1^ GdSi_PEG_ stable colloid in glucose 5.5% was perfused into the catheterized tail vein of healthy male Sprague-Dawley rats as a bolus (0.04 mmol Gd kg^−1^). Subsequently, *T*_1_- and *T*_2_-weighted images were acquired before (baseline) and after the administration of the CA by using a 7 T horizontal scanner. 

**Figure 8:**
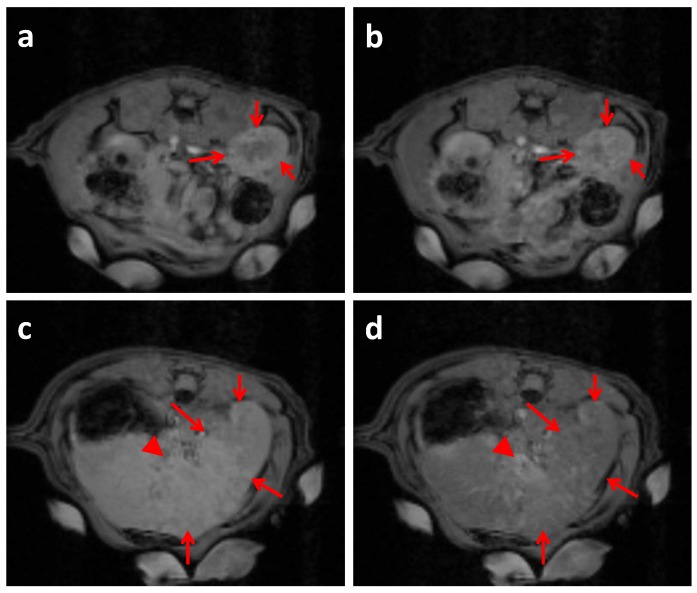
*In vivo* coronal *T*_1_-weighted images acquired from a male Sprague-Dawley rat at 7 T magnetic field. (**a**,**c**) Control (baseline) with no MNP administration; (**b**,**d**) Acquisition 30 min after GdSi nanoparticles injection (0.04 mmol Gd^3+^ kg^−1^); Red lines show up kidneys (**a**,**b**) and liver (**c**,**d**).

Figure 8 shows coronal *T*_1_-weighted images depicting kidneys (Figure 8a,b, red arrows) and liver (Figure 8c,d, red arrows), respectively, before (Figure 8a,c) and after (Figure 8b,d) CA administration. These organs receive most of the blood stream showing highly significant particle accumulation, which promotes a noticeable MRI *T*_1_ contrast enhancement. Kidneys showed a 7% increase in the MRI signal intensity (SI) after the CA administration. Liver presented non-homogenous changes, probably due to restricted nanoparticle biodistribution and pharmacokinetics, with some areas depicting large changes up to 21% (Figure 8c,d, arrowhead), whereas other areas showed no changes or even a decrease in SI. The contrast enhancement in the liver stands for at least one hour, the time we followed the contrast agent by MRI. We can hypothesize that contrast will be enhanced for longer because when we stopped the acquisition we were still able to detect a signal intensity enhancement with respect to baseline.

We are aware that similar MRI *T*_1_ contrast increase has also been recently reported with other paramagnetic nanoparticles [48,49]. However, the GdSi system shows improved stability and a superior safety profile in biological fluids, as no cation leaching was observed in dissolution tests carried out for 24 h. Moreover, although this is not the aim of this work, in the case of application to tumor development study, GdSi nanoparticle delivery to cancer cells may be promoted by surface incorporation of specific moieties that target upregulated peptides in rapidly-dividing cells [50]. 

Conversely, despite the good MRI *in vitro* results formerly presented for *T*_1_, we must say that no significant change was observed in *T*_2_ weighted images following CA administration.

## 3. Experimental Section

### 3.1. Materials 

All reagents were purchased from Sigma-Aldrich (St. Louis, MO, USA), except Gd(NO_3_)_3_·6H_2_O (ABCR, Karlsruhe, Germany) and HPLC grade solvents (Scharlab, Barcelona, Spain). Gd-Si oxide nanoparticles protected with a PEG external coating were synthesized in a three-step process (Figure 1).

HeLa cell line, D6-3T3 (fibroblasts cells), 42-MG-BA (glioblastoma multiforme cells), and SH-SY5Y (neuroblastoma cells), were purchased from the German Collection of Microorganisms and Cell Cultures, Braunschweig, Germany. Reagents used for cells growth were MEM Eagle, RPMI Medium 1640, DMEM, and Ham (F12) Nut MIX (Gibco BRL-Life Technologies, CA, USA) fetalbovine serum (FBS), and penicillin/streptomycin solution (Pen-Strep). Dimethyl sulfoxide (DMSO) and 3-(4,5-dimethylthiazol-2-yl)-2,5-diphenyltetrazoliumbromide (MTT) were purchased from Sigma-Aldrich (St. Louis, MO, USA).

Male Sprague-Dawley rats (250–300 g) (three specimens) were acquired from Janvier Labs (France) and maintained under a 12/12-h light/dark cycle (lights on 07:00–19:00 h) at room temperature (22 ± 2 °C), with free access to food and water. Rats were housed in group and adapted to these conditions for at least one week before experimental manipulation. All experiments were approved by the local authorities (CSIC-UMH) and were performed in accordance with Spanish (law 32/2007) and European regulations (EU directive 86/609, EU decree 2001-486).

### 3.2. Synthesis of Gd(H_2_O)_4_[Fe(CN)_6_]

Firstly, we prepared the (Et_4_N)_3_[Fe(CN)_6_] precursor following a known recipe [51]. Briefly, 3.3 g (10 mmol) of K_3_[Fe(CN)_6_] and 6.3 g (30 mmol) of Et_4_NBr were dissolved with 200 mL of methanol in a 500 mL flask connected to a nitrogen line, and stirred at 30 °C for three days under N_2_ atmosphere. The mixture was filtered off and the filtrate was concentrated to approximately 10 mL by rotary evaporation. The resulting solution was stirred with 100 mL of ethyl ether and the yellow precipitate was collected by filtration. The crude was dissolved in 150 mL of refluxing acetonitrile and the solution was allowed to cool, obtaining (Et_4_N)_3_[Fe(CN)_6_]. For nanosized Prussian Blue analogue preparation, Gd(NO_3_)_3_·6H_2_O (0.86 g, 2.5 mmol) were dissolved in 50 mL of EtOH:H_2_O (2.5:1 *v*/*v*). Then, a solution of the previously-synthesized (Et_4_N)_3_[Fe(CN)_6_] (1.5 g, 2.5 mmol, in 15 mL methanol) was added and the mixture was left for a day at room temperature. The precipitate was filtered off, washed with ethanol, and vacuum dried to yield 118 mg of an orange powder (GdFe).

### 3.3. Synthesis of GdSi Oxide/hydroxide Nanocomposite

Dense, Gd(OH)_3_·3SiO_2_·*x*H_2_O nanoparticles (GdSi) were prepared by modification of a previously reported method [40,41]. Gd(H_2_O)_4_[Fe(CN)_6_] (550 mg, 1.25 mmol), was suspended in 278 mL of EtOH:H_2_O (2.5:1 *v*/*v*) with vigorous stirring. Then, 3-cyanopropiltrichlorosilane (697 µL, 4.50 mmol) was added and, after 30 min, 3.5 mL ammonia solution and 11 mL of sodium silicate solution (0.54% SiO_2_, 2.69 mmol) were introduced. The reaction was allowed to proceed for 48 h. Non-reacted silicate ions were removed by subsequent centrifugation (484 g, 2 h), and the obtained white solid was washed repeatedly with milliQ water, centrifuged again (1935 g, 30 min), and further freeze dried (−55 °C, 16 h).

In order to modify the GdSi surface, we used a method previously described by our group [45]. 375 mg of Gd(OH)_3_·3SiO_2_·*x*H_2_O was dried at 80 °C and vacuum (8 torr) for 24 h. Afterwards, 7.4 mL of anhydrous toluene was added and the mixture was heated to reflux. Then, 728 µL (3.13mmol) of 3-aminopropyltriethoxysilane (APTES) was added and the mixture was stirred for 3 h. The obtained product was filtered off, washed with toluene and methanol and freeze-dried (−55 °C, 16 h). Next, 156 mg of the above material was suspended in 15.6 mL of anhydrous dichlorometane. Then, 195 µL of diisopropyl amine were injected under nitrogen atmosphere. Afterwards, 234 mg of 2,5,8,11-tetraoxatetradecan-14-oic acid succinimidyl ester (PEG_4_) was added. The reaction was stirred overnight at room temperature. Later, the solvent was removed under reduced pressure and the nanoparticles were suspended in 100 mL of ethanol with stirring. Subsequently, the suspension was filtered off and washed with ethanol (300 mL). Finally, the material (GdSi_PEG_) was freeze-dried (−55 °C, 16 h). 

### 3.4. Materials Characterization

Powder X-ray diffraction (XRD) patterns were collected in a Philips X’Pert diffractometer equipped with a graphite monochromator, operating at 40 kV and 45 mA and using nickel-filtered Cu K_α_ radiation (*λ* = 0.1542 nm). Liquid nitrogen adsorption isotherms of 200 mg of sample were measured in a Micromeritics Flowsorb apparatus. Surface area calculations were done by the BET method. Samples for transmission electron microscopy (TEM) were ultrasonically dispersed in a mixture of EtOH:H_2_O (2.5:1 *v*/*v*) and transferred to carbon-coated copper grids. TEM and scanning transmission electron microscopy (STEM) micrographs were collected in a JEOL JEM 2100F microscope operating at 200 kV. The quantitative energy-dispersive X-ray spectroscopy analysis (EDS) was performed in an INCA Energy TEM 250 system from Oxford Instruments, working with a SDD X-MAX 80 detector. Field-emission scanning electron microscopy (FESEM) micrographs were collected in a ZEISS Ultra 55 microscope operating at 2 kV, with a 2 × 10^−9^ A beam current and 2.5 mm as the working distance. Infrared spectra were recorded at room temperature in the 400−3900 cm^−1^ region with a Nicolet 205xB spectrophotometer, equipped with a Data Station, at a spectral resolution of 1 cm^−1^ and accumulations of 128 scans. Samples were outgassed at 400 °C under vacuum during 1 h for complete water removal before acquisition. Finally, water and organic (PEG_3_) content were calculated from elemental analysis (FISONS, EA 1108 CHNS-O) and thermogravimetric (TGA) measurements (Mettler-Toledo TGA/SDTA851_e_).

### 3.5. Stability Assays

GdSi_PEG_ material stability in physiological medium was monitored by solution assay in phosphate-buffered saline (PBS, 1×) or glucose aqueous solution (5.5 wt. %). 5 mg of the corresponding material were stirred in 1 mL at 1500 rpm and 37 °C in a Thermomixer^®^ for 24 h. Then, samples were centrifuged (16,100 *g*, 15 min) and supernatant metal cation concentration was analyzed by inducted couple plasma (ICP).

### 3.6. Cell Biocompatibility

The different cell lines were cultured in 96-well cell culture plates with the seeding densities shown below in a final medium volume of 200 µL/well: HeLa 10,000 cells/mL; fibroblast 3T3 and glioblastoma 42-MGBA 50,000 cells/mL and neuroblastoma SH-SY5Y 100,000 cells/mL. HeLa cells were grown in MEM (Earle’s), 3T3 cell line in DMEM, 42-MGBA cell line in MEM (Earle’s) and RPMI 1640 (1:1), and SH-SY5Y cell line was cultured in a DMEM and Ham (F12) Nut MIX (1:1). Cell media were supplemented with 10% FBS and Pen-Strep 1:100 (*v*/*v*). The plates were cultured 24 h at 37 °C and 5% CO_2_ injection in air. After 24 h cells were treated with the CA at a concentration range of about 0.25–100 µg/mL in RPMI medium. Cells with nanoparticles were incubated at 37 °C for 24 h and 5% CO_2_. All concentrations were tested in triplicate and negative controls with no nanoparticles were also carried out.

Cell viability was measured using the 3-(4,5-Dimethylthiazol-2-yl)-2,5-diphenyltetrazolium bromide (MTT) assay. Briefly, 200 µL/well of MTT/PBS (1 mg/mL) were added and the plates were incubated at 37 °C for 3 h. Formazan crystals were dissolved with 100 µL DMSO and then absorbance at 595 nm was measured with a 1681130 iMark^TM^ Microplate Reader. Absorbance values were normalized with respect to the control and expressed in percentage using the next Equation (4):

(4) $$Relative\;cell\;viability\;=\;\frac{OD_{595\;TEST\;SAMPLE}}{OD_{595\;CONTROL}}\;\times 100$$

Data statistical analysis was performed applying arithmetic’s means and error bars of statistical error means (SEM) using MATLAB (MathWorks).

### 3.7. ^*1*^H NMR Relaxometry Study and In Vitro MRI.

Relaxivity determinations and MRI studies were carried out in a clinical 3 T MRI unit (Philips Achieva 3 T X-Series; Philips Healthcare, The Netherlands) with an eight-channel phased array head coil. The phantom was scanned with a single slice in the coronal orientation, obtaining a transversal view of all tubes filled with variable CA concentration. Geometric parameters remain equivalent between the *T*_2_ and *T*_1_ estimation sequences (field of view 220 × 220 mm^2^, slice thickness 5 mm and 1.0 × 1.0 mm^2^ in plane resolution) sharing the same spatial localization. A multi-echo spin echo sequence was used to estimate *T*_2_ values acquiring 32 echoes ranging from 14 to 231 ms (Δ*TE* = 7 ms) with a sequence *TR* of 2000 ms. *T*_1_ values of each tube were estimated using a look-locker inversion recovery acquisition with 107 inversion times ranging from 6.51–5306.51 ms, with an inversion time interval of 50 ms. A new inversion pulse was applied every 6 s to avoid signal saturation due to extremely close inversion pulses. To reduce the influence of readout excitation pulse in the final *T*_1_ values, an excitation flip angle of 5° was applied during the TFE shot [47]. With regards to image analysis, *T*_2_ maps were generated by nonlinear fitting of the signal acquired at every *TE* to a mono-exponential model. The same procedure was applied to every image pixel producing *T*_2_ maps. Moreover, *T*_1_ maps were generated by nonlinear fitting of the signal acquired at every inversion time to the signal model described at [52]. As in the case of *T*_2_ maps, the same procedure was applied to every image pixel to generate the final *T*_1_ map. Quantitative values were obtained in aqueous xanthan gum (0.1%) nanoparticle suspensions by manual region of interest (ROI) analysis over *T*_2_ and *T*_1_ maps. A circular ROI was placed for each tube, avoiding the border. In each ROI, means and standard deviations were computed for further comparison. The resulting *T*_1_ and *T*_2_ values were averaged and plotted as 1/*T_i_* (s^−1^) where *i* = 1, 2 *versus* [Gd^3+^] (mM), the slopes of these graphs provided the specific relaxivities *r*_1_ and *r*_2_. 

### 3.8. In Vivo MRI Studies Imaging

Rats were anesthetized in an induction chamber with 3%–4% isofluorane in medical air (0.8–1 L/min) and maintained with 1%–2% isofluorane (IsoFlo) during the MRI experiment. Anesthetized animals were taped down in a custom-made animal holder to minimize breathing-related movement artifacts. The body temperature was kept at 37 °C using a water blanket and animals were monitored using a MRI-compatible temperature control unit (MultiSens Signal conditioner, OpSens, Quebec, Canada). The breathing rate was also measured using a custom designed device. Experiments were carried out in a horizontal 7 T scanner with a 30 cm diameter bore (Biospec 70/30v, Bruker Medical, Ettlingen, Germany). The system had a 675 mT/m actively-shielded gradient coil (Bruker, BGA 12-S) of 11.4 cm inner diameter. A ^1^H rat body receive-transmitter resonator (Bruker BioSpin MRI GmbH, Germany) was employed. Data were acquired with a Hewlett-Packard console running Paravision software (Bruker Medical GmbH, Ettlingen, Germany) operating on a Linux platform. 

Studies were performed by injecting 1 mL of the CA suspension (5 mg·mL^−1^) into the catheterized tail vein as a single bolus (0.04 mmol Gd/kg body weight), and acquiring images 30 min afterwards. Acquisition was held for 1 h. *T*_2_-weighted anatomical images to position the animal were collected in the three orthogonal orientations using a rapid acquisition relaxation enhanced sequence (RARE), applying the following parameters: field of view (FOV) 40 × 40 mm, 15 slices, slice thickness 1 mm, matrix 256 × 256, effective echo time (*TE*_eff_) = 56 ms, repetition time (*TR*) = 2 s, 1 average and a total acquisition time of 64 s [53,54]. Two types of images were used to assess the effect of CA in the signal intensity in *T*_1_- and *T*_2_-weighted images. For the former, FLASH images were acquired with the following parameters: 25 slices, 1.5 mm slice thickness, *TR* = 197 ms; *TE* = 2.7 ms; FOV = 6.0 × 5.0 cm; Mtx = 128 × 108; averages = 4; total acquisition time = 90 s. Three images were acquired before the CA administration (baseline) and 20 after it. For the *T*_2_-weighted images, RARE was used with the same geometry than the *T*_1_-weighted images and the following parameters: *TR* = 2800 ms; *TE* = 48 ms; FOV = 6.0 × 5.0 cm; Mtx = 256 × 214; averages = 8; total acquisition time = 600 s. One of these images was acquired at the beginning (baseline) and one at the end of the experiment. Data were analyzed with Image J (W. S. Rasband, U.S. National Institutes of Health, Bethesda, MD, USA).

## 4. Conclusions

Gd-Si oxide nanoparticles obtained by alkaline transformation of a Prussian Blue analogue are a novel MRI contrast agent with higher longitudinal and transversal *in vitro* relaxivity values than commercial Gd^3+^ chelate solutions, excellent stability in physiological fluids, no significant cytotoxicity, and easy surface functionalization. These properties prove their potential as a valid alternative to the current CA systems used at the clinical stage, mostly in those cases where the poor pharmacokinetics profile and potential toxicity of Gd^3+^ soluble forms can become an issue (e.g., patients with renal failure). In this context, future studies will be focused on long-term *in vivo* stability and potential toxicity of this novel paramagnetic nanocomposite.

## Figures and Tables

**Table 1:** Compositional and textural characteristics of as-synthesized materials.

Sample	Molecular Formula	Atomic Composition (%) *^a^*			Size (nm)		Area BET (m^2^·g^−1^) *^b^*
		Gd	Fe	Si	TEM *^c^*	DLS *^d^*	
GdFe	Gd(H_2_O)_4_[Fe(CN)_6_]	52.8	47.2	--	94.2 ± 35.5	nd *^e^*	nd *^e^*
GdSi	Gd(OH)_3_·3SiO_2_·*x*H_2_O	18.6	1.3	80.1	75.2 ± 24.0	84 ± 44	19.0

*^a^* As determined from the energy-dispersive X-ray spectroscopy analysis (EDS) study; *^b^* Brunauer-Emmett-Teller (BET) surface area analysis; *^c^* Avg ± SD, as determined by transmission electron microscopy (TEM) measurement of at least 200 particles; *^d^* Diffuse light scattering (DLS); *^e^* nd = not determined.

**Table 2:** Relaxivity properties for sample GdSi sample and commercial gadopentetate dihydrogen salt (Gd-DTPA).

Sample	*r*_1_/s^−1^·mM^−1^	*r*_2_/s^−1^·mM^−1^	*r*_2_/*r*_1_
GdSi	5.6	8.2	1.4
Gd-DTPA	4.1	3.3	0.8
			
